# University of Nashville

**Published:** 1857-06

**Authors:** 


### UNIVERSITY OF NASHVILLE.

In the last number of our Journal, we intimated that an impression existed upon our part, that the Faculty of the Nashville School had acted in bad faith towards the Atlanta Medical College, in requiring the withdrawal of Prof. Buchanan, from the position which he occupied in the latter institution.

At a recent conference, however, with some of the gentlemen connected with the Nashville College, we have had facts and assurances satisfactorily supplied, which enable us to relieve *the Faculty*, from the imputation of any improper motive or dishonorable intention in connection with that matter.

